# Causality and scientific explanation of artificial intelligence systems in biomedicine

**DOI:** 10.1007/s00424-024-03033-9

**Published:** 2024-10-29

**Authors:** Florian Boge, Axel Mosig

**Affiliations:** 1 Bioinformatics Group, Department for Biology and Biotechnology, Ruhr-University Bochum (RUB), Gesundheitscampus 4, 44801 Bochum, NRW Germany; 2 Institute for Philosophy and Political Science, Technical University Dortmund, Emil-Figge-Str. 50, 44227 Dortmund, Germany; 3 Center for Protein Diagnostics, Ruhr University Bochum, Gesundheitscampus 4, 44801 Bochum, Germany

**Keywords:** Explainable artificial intelligence, Scientific explanation, Trustworthiness

## Abstract

With rapid advances of deep neural networks over the past decade, artificial intelligence (AI) systems are now commonplace in many applications in biomedicine. These systems often achieve high predictive accuracy in clinical studies, and increasingly in clinical practice. Yet, despite their commonly high predictive accuracy, the trustworthiness of AI systems needs to be questioned when it comes to decision-making that affects the well-being of patients or the fairness towards patients or other stakeholders affected by AI-based decisions. To address this, the field of explainable artificial intelligence, or XAI for short, has emerged, seeking to provide means by which AI-based decisions can be explained to experts, users, or other stakeholders. While it is commonly claimed that explanations of artificial intelligence (AI) establish the trustworthiness of AI-based decisions, it remains unclear what traits of explanations cause them to foster trustworthiness. Building on historical cases of scientific explanation in medicine, we here propagate our perspective that, in order to foster trustworthiness, explanations in biomedical AI should meet the criteria of being scientific explanations. To further undermine our approach, we discuss its relation to the concepts of causality and randomized intervention. In our perspective, we combine aspects from the three disciplines of biomedicine, machine learning, and philosophy. From this interdisciplinary angle, we shed light on how the explanation and trustworthiness of artificial intelligence relate to the concepts of causality and robustness. To connect our perspective with AI research practice, we review recent cases of AI-based studies in pathology and, finally, provide guidelines on how to connect AI in biomedicine with scientific explanation.

## Introduction

**Fig. 1:**
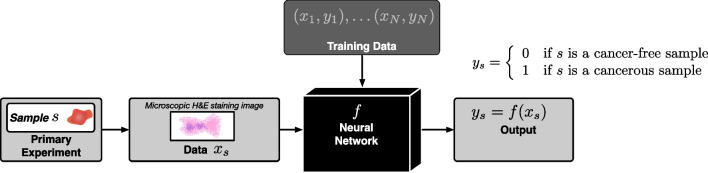
Illustration of supervised learning. Here, pathology images ($x_i$) are annotated with labels as cancerous or cancer-free ($y_s$). A classifier is a mathematical function that takes an image as an input, and outputs a label. The classifier is trained using training data, consisting of a collection of labelled images

While many deep learning-based artificial intelligence (AI) systems are well recognized for their high predictive accuracy, their robustness and trustworthiness are often put into question when they are involved in high-stakes decisions, in particular in medical diagnosis. These systems are often trained in a supervised manner on substantial amounts of training data, as illustrated in Fig. 1, and in order to address these questions of trustworthiness and robustness, the need for explaining AI systems and the output they produce has been emphasized. As reflected by many authors [26, 40], the explanation of biomedical AI systems commonly seeks to answer the key questions of what deep learning models have learned and how they arrive at their decisions, where addressing the lack of trustworthiness is commonly the central issue. The cause of this lack is commonly seen in the opacity or black-box nature of neural network-based approaches [31], which has triggered the emergence of eXplainable AI, or XAI for short, as an important subdiscipline in the AI research field. As many XAI approaches aim to uncover internal representations of the underlying data, some authors have suggested to interpret or understand these representations in the light of causality [23].

The goal of this contribution is to bring in line two aspects of explanation and causation: On the one hand, explanation and causation have taken important roles as warrants of progress in the history of biomedicine, while on the other hand, they now take important, albeit not yet well understood, roles in the context of artificial intelligence. In this contribution, we seek to connect the historical roles of explanation and causation with their new roles in the context of AI. To establish this perspective, our contribution is organized as follows. We start with discussing prominent historical cases where scientific explanation and formal concepts of causation have had an impact on biomedicine. In the “Formal concepts of explanation and causality” section, we then review formal concepts and approaches that address questions of explanation and causation in medical AI. The “Scientific explanation, robustness, and trustworthiness” section discusses how these concepts and approaches affect the robustness and trustworthiness of the underlying AI systems. Finally, the “Case studies in current biomedical AI” section briefly reviews how explanation and causation have been accounted for in recent literature in the field of computational pathology. Our contribution addresses biomedical researchers as well as medical practitioners, many of whom may perceive a tension between biomedicine and AI research: On the one hand, the key driver of progress in medical research over the past decades has been science, particularly aiming at the explanation and understanding of diseases and their treatments. Now, on the other hand, opaque and often inscrutable machine learning models are being discussed as taking a role in decision-making processes in biomedicine [15]. From this perspective, our present contribution aims to help understand this tension, and potentially identify future directions along which this tension can be loosened.

Explainable artificial intelligence is often approached and discussed from a methodological perspective, where the focus is on computational methods that derive interpretable output from predictive deep neural networks. Computational methods, however, provide no basis for answering the guiding question of what traits of explanations foster trustworthiness and robustness. Here, ideas from the philosophy of science, or epistemology, come into play, which has dealt with related questions over the past decades. Consequently, the first part of our survey includes an overview of the relevant contributions from the field of epistemology.

## Historical case studies of explanation and causality in biomedicine

The importance of explanation in medicine is intertwined with the role of causality, as is well supported by historic examples. Among the most prominent cases may be the identification of lemons as a remedy against scurvy by James Lind in 1753 [30], and the discovery of vitamin C as the scientific explanation of the remedy. For the role of causality, an illustrative historical case is provided by smoking as a cause of lung cancer, which was a matter of debate among statisticians throughout the twentieth century. In this section, we will use these historical cases for introducing basic concepts and motivating key questions.

### Scientific explanation in medicine

In what is often considered the first randomized controlled clinical trial (RCT), James Lind demonstrated that the inclusion of lemons into the diet of sailors provides a remedy against scurvy. Although Lind’s RCT provided good evidence that lemons are an effective treatment, another half century passed before the findings were translated into action. Even then, the remedy remained disputed for more than a century to follow, and many questions emerged that can be attributed to a *lack of explanation*: Why did lemon juice become ineffective after boiling at high temperatures or after contact with copper? Why were lemons more effective than limes? Why did rats not suffer from scurvy, while guinea pigs did? The adoption of the citrus remedy, as documented by Harvie [20], was accompanied by repeated unexplained failures, which put it under debate over again.

Questions were finally resolved with the discovery of vitamin C as the antiscorbutic factor by Szent-Györgyi and Haworth [47], and the subsequent elucidation of its role in the biosynthesis of collagen. This scientific explanation once and for all concluded the debate about the treatment and prevention of scurvy and provided scientifically testable answers to the previously unanswered questions. We here suggest that the historical example of scurvy makes the case for the importance of scientific explanation in medicine: Although RCTs establish a causal relationship between a particular treatment and the cure of a specific disease, questions emerge after the treatment is adopted into medical practice, and leaving these questions unanswered undermined its trustworthiness.

Explanation is also considered of importance in the context of drug effectiveness, where the role of explanation is taken by a mechanism of action [34]. In fact, most, although by far not all, drugs that are administered in modern medicine are explained by an underlying mechanism of action. One prominent example of a drug without a known mechanism of action is lithium [37], whose effectiveness for treating bipolar disorder is supported by randomized clinical trials [11]. Among a list of potential mechanisms, it remains unclear for each of these mechanisms whether it contributes to the effectiveness, or rather is a cause of side effects. The contrast between the well-explained case of vitamin C and the unexplained case of lithium illustrates how scientific explanation yields insights that eventually improve the treatment in ways that would not have been possible if left unexplained. In short, mechanisms of action are inherently scientific explanations that are usually experimentally testable in manifold ways, and they resolve uncertainties towards treatment efficacy, and hence undermine trustworthiness.

## Formal concepts of explanation and causality

The relevance of mechanisms of action as explanations of drug efficacy relates back to the core question: Can scientific explanation improve the trustworthiness of AI systems, and how does scientific explanation relate to AI? Before we look for answers to these questions in the “Formal concepts of explanation and causality” section, it is important to review essential concepts behind randomized controlled trials (RCTs) as the basis for judging the efficacy of medical treatment. As RCTs determine whether a cause-and-effect relationship exists between a treatment and the cure of disease, they are related to important concepts of causation in statistics. Causation precedes explanation in the sense that it is obviously futile to consider mechanisms of action for treatments without causal efficacy, and it can be argued that the same precedence holds for AI systems: AI models should be causal for treatment efficacy when they are involved in treatment decisions, and only after efficacy is established, it makes sense to raise questions of explanation. As the concepts of causation and explanation are related, both in science in general and in machine learning specifically, we first review basic concepts of causation before turning to matters of explanation.

### The *do*-calculus and causality

Cause and effect are key concepts in the conduct of science, and have correspondingly been the subject of investigation and debate among both philosophers and practicing scientists. One of the prominent debates about causality in medicine circled around the question whether smoking causes lung cancer, as accounted in detail by Judea Pearl [39]. The causal effect of smoking on developing lung cancer was fiercely rejected by the statistician Ronald Fisher, who used much of his scientific authority to uphold that the undeniable correlation between smoking and cancer by no means implies causation. The resulting debate of distinguishing correlation from causation is indeed a key problem that has been the subject of debate among statisticians throughout the twentieth century. Due to groundbreaking work on causal inference [38], there is now a widely accepted consensus on how to establish causality between two variables, which dates back to the work of Sewall Wright [51]: to undermine the claim that *A* (smoking) causes *B* (cancer), one needs to conduct an intervention experiment: A representative population needs to be recruited. This population is randomly subdivided, with one half undergoing intervention *A*, and the other half undergoing no intervention, so that the probability of effecting *B*, formally written as $p(B|\textrm{do}(A))$, can be determined. This so-called do-calculus has been comprehensively investigated [38] and can be regarded as the statistical foundation of RCTs as the gold standard for determining the effectiveness of medical treatment.

### The ladder of causation

We have now encountered two steps on the ladder of causation, as proposed by Pearl [39]: The first step is mere *association*, as can be established by conventional statistical tools, e.g., by measuring the correlation between two variables. The second level, as constituted by the plain *do*-calculus involving two variables, is the level of *intervention*, which requires experimental interference by altering one variable and observing the consequence on another variable.

**Fig. 2:**
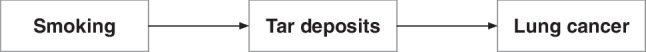
*Example of a causal diagram* [39]. For establishing the causality between smoking and lung cancer, the deposition of tar in the lung can be introduced as an intermediary variable. With the help of this intermediary variable, evidence for the causality of smoking to lung cancer can be obtained without conducting an ethically prohibitive randomized intervention study on the smoking status

The third step on the ladder of causation is *counterfactuality*, which involves determining probabilities in unobserved what-if scenarios. In such a what-if setting, the do-calculus unfolds its complete power when dealing with more than two random variables. In fact, causal inference often naturally involves more than two variables, as the smoking-causes-cancer example illustrates in Fig. 2: Here, a third variable of *tar deposits* is taken into consideration, reflecting the causal chain that smoking causes tar deposits in the lung, and these deposits in turn cause lung cancer. Such causal chains, and more complex ones with more variables, can be depicted as a causal diagram, where the variables are indicated by vertices, and a hypothesized causality between two variables as a directed edge between the corresponding vertices. Given a hypothesized causal diagram, the do-calculus provides means to investigate counterfactuals, i.e., what-if scenarios resulting from intervening with certain variables in the diagram. In particular, the do-calculus provides means to compute probabilities of such counterfactuals, which is particularly relevant when direct intervention is not possible. For example, it is infeasible to conduct an RCT that intervenes on the smoking status of human participants. As illustrated in Fig. 2, introducing an intermediary variable that represents the accumulation of tar deposits can overcome the difficulty to establish causality by first collecting causal evidence that smoking causes tar deposits, and then collecting evidence that tar deposits cause cancer. The diagram in Fig. 2 is only a simple example, and the methodological toolbox of causal inference allows to infer causal relationships and the computation of counterfactual *what if* probabilities for much more complex constellations of random variables.

This third step of counterfactuality on the ladder of causation is in fact of utmost relevance for causal inference in practice. In many settings, for example, in the case of smoking as a cause of cancer, direct intervention on the causal variable is impossible or prohibitive in practice. In such cases, counterfactuals from a suitable causal diagram can be taken into account to obtain the relevant probabilities in terms of the do-calculus. For the smoking-causes-cancer example, a suitable causal diagram may involve further variables beyond tar deposits in the lung, such as factors of genetic predisposition.

### Explainability approaches in artificial intelligence

The need for involving explainable AI is often motivated by the problem of model generalization. AI models in biomedicine are trained on data obtained from one or few clinical trials, or acquired in one or few laboratories or measurement devices. Since each clinical trial, laboratory, or measurement device produces data with its own distinct bias, the predictive accuracy of AI systems significantly drops when they are evaluated by data from a different clinical trial, laboratory, or measurement device that was not involved in the training data. From a machine learning perspective, one thus aims for AI systems that ignore variances that are due to biased confounding factors and inhibit generalization performance. Rather, one aims to devise AI systems that focus on those variances that are due to biomedically relevant phenomena. One prominent example of a biased AI system has been reported in Zech et al. [52], where the authors demonstrated that an AI system for identifying pneumonia in a multi-centric X-ray study learned to identify data signatures obtained from an emergency room device, where the prevalence of pneumonia was significantly higher. While improving performance on the given data, this clearly inhibits performance on data from other clinics, where the particular signature will not be present. In the light of this example, the motivation of involving XAI approaches is to determine whether a decision of an AI system was based on a confounding variance, or obtained from identifying a biomedically plausible feature of disease. As we discuss in this section, the terms explainability and explanation in the context of XAI lack a unique unambiguous defintion, and are not well connected to the notion of explanation in science that we saw in the “Historical case studies of explanation and causality in biomedicine” section. Throughout this section, we correspondingly use these terms loosely defined, while in the “Scientific explanations for machine learning models” section, we will identify how the traits of scientific explanation can be merged with concepts from XAI.

A dominant approach in the field of XAI are so-called *post-hoc* explainability approaches: First, an AI model is trained and evaluated for predictive accuracy on a given set of data for training and validation, while it is left as a second and subsequent step to derive explainable information from the model and the output derived from it. As reviewed in Guidotti et al. [17], there is a vast amount of literature on computational methods that support this post hoc approach. The way in which such post-hoc methods commonly work is best illustrated by an image classification model. A deep neural network or other machine learning model is trained to take an input image of a certain size and classify it into different classes, e.g., *cancerous* vs. *cancer-free*. Now, provided an image to be classified along with a readily trained machine learning model, a post hoc explainability algorithm will provide an additional output that indicates, for each pixel of the input image, how relevant this particular pixel was for arriving at the classification of the whole image. This localized relevance information yields a heatmap that provides interpretable information about the inner workings of the machine learning model. This heatmap approach constitutes the broad class of post hoc explainability approaches, which, given an input data point, assign an intensity to each input variable according to how relevant the variable was for producing its specific output. Visualizing these input relevances yields the heatmaps as an explainable model output.

While the heatmaps provided by post-hoc (and other) XAI methods are indeed interpretable and explainable and may be helpful to identify whether an AI output was based on a confounding feature or a biomedically relevant disease characteristic, they somewhat avoid to commit to an explicit notion of what actually constitutes a valid explanation, and how the explanation fosters trustworthiness.

Often, the role of an explanation is expert-centric in the sense that the explainable output is to be assessed by a human expert [1] or other human stakeholders [8]. Not surprisingly, this expert-centrism has been criticized [15], since heatmap-based visualization may be misleading [25] or their interpretation ambiguous [15]. The criticism of expert-centric explanation essentially leaves us with two directions to follow: either abandon the explanation of AI models overall and accept their black-box nature or, alternatively, establish explanations that are not expert-centric by asking the question of what constitutes a legitimate explanation of an AI model. Recently, Wang et al. [49], building on the framework from Schuhmacher et al. [41] discussed in detail below, have identified the five aspects of *localization*, *visual recognizability*, *physical attribution*, *model transparency*, and *actionability* as the relevant aspects of explainability. In a similar direction, Nauta et al. [35] distill 12 properties from an extensive review of explainable AI literature.

Besides heatmaps, or more generally, the computation of importance values for the input variables of machine learning models, several approaches have been devised that involve the concept of causality to foster the explainability of AI models. Among the earliest approaches in this direction are *counterfactual* explanations, as originally proposed by Wachter et al. [48]. In their sense, a counterfactual explanation for a given input image is a minimal modification of the input that would change the classification outcome. The minimally modified input is referred to as the counterfactual data point. An attractive trait of this approach is that it provides a scientific explanation that is, at least in theory, experimentally testable: One could attempt to alter an object to represent the counterfactual data point and experimentally test whether this changes the class (e.g., the disease status) as which the object is to be classified. If such an experiment would uncover that the suggested counterfactual modifications, which were derived using the AI model, lead to a change in class on a real-world object, one would indeed have obtained experimental counterfactual evidence for the correctness of the AI model’s output. While this clearly substantiates the trustworthiness in the model, conducting counterfactual modifications is usually severely restricted in practice. For medical imaging data, one can obviously not produce samples or patients that realize an altered morphology in some part of a medical image. Also for molecular data, it will generally be impossible to alter one specific genetic or metabolic factor, or even the age or demographic background of a patient. Some attempts have been made to make counterfactual explanations more realistic through so-called algorithmic recourse [24], though it remains to be seen where these approaches can be realized in practical applications.

Counterfactual explanations have been reviewed extensively by Chou et al. [10]. As Chou et al. find, counterfactual explanations proposed in the literature to date fall short of the formal and structured theory of causality described in the “The ladder of causation” section. Thus, their review arrives at the somewhat sobering conclusion that *these systems cannot promote a causal understanding to the user without the risk of the explanations being biased, sub-optimal, or even erroneous*. This has been addressed in recent work by Baron [2] as well as Buijsman [5], who propose theoretical frameworks to connect counterfactual explanations with the formal theory of causation outlined in the “The ladder of causation” section. One can summarize that in the current state of research, counterfactual explanations are sometimes practically useful, but at least in their theoretically sound form are difficult to realize in practical settings.

**Fig. 3:**
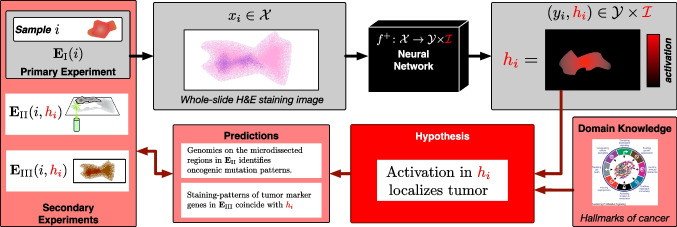
Framework for falsifiable explanations in artificial intelligence (FXAI). We assume that data are collected for a set of identifiable objects or entities. The data processed by a neural network are obtained by conducting a primary experiment $\textbf{E}_{\textrm{I}}(i)$ on an object *i*. The network $f^+$ is trained in a supervised manner to identify labels in a domain $\mathcal {Y}$, and additionally produces interpretable output $h_i$. For image data, $h_i$ is often a heatmap. The actual explanation is a scientific hypothesis (red box) that connects $h_i$ with the original sample *i*, so that the hypothesis can be tested experimentally through various secondary experiments $\textbf{E}_{\textrm{II}}$, $\textbf{E}_{\textrm{III}}$, $\dots$

### Scientific explanations for machine learning models

The question of what constitutes a legitimate explanation of an AI model and its output brings us to the central topic of this manuscript. More precisely, we focus on the question of legitimate *scientific* explanation for a simple reason: With modern medicine built on science, why should we impose much weaker standards on the science of AI, especially in high-stakes contexts of use, wherein we should require evidence for trustworthiness? From a scientific explanation perspective, it becomes immediately clear that expert-centrism is to be avoided, since in science, we do not rely on expert judgement as the ultimate arbiter for the quality of an explanation. Rather, proposed explanations have to face the tribunal of experience in order to count as scientific and credible [12, 21]. In other words, explanations need to have testable consequences. Current practice in XAI, in which explanations are typically judged by experts, thus falls short of regular standards of scientificality. Correspondingly, the primary goal of our manuscript is to establish a notion of XAI that is in line with scientific explanation.

Supposing a scientifically sound notion of XAI, a second key question arises: How does explanation foster the trustworthiness of machine learning systems? This question also falls in the scope of our present contribution. As we will argue, some core problems of XAI arise from how ML and its outcomes relate—or fail to relate—to evidence about physical reality. As such, the predominant problems are not exclusively computational but of a fundamental, philosophical nature. Putting together the need for explanation in medical AI on the one hand, and the importance of scientific explanation for trustworthiness in medicine on the other, it appears almost inevitable to ask for combining both sides: can we explain machine learning models in a scientific manner?

Recently, Schuhmacher et al. [41] introduced a framework for *falsifiable explanations* in the context of medical AI, to be referred to as FXAI in the sequel. The FXAI framework, in fact, provides a formal definition of explanation: As illustrated in Fig. 3, the framework first and foremost distinguishes explicitly between a sample *s* as part of physical reality, which is uniquely identified through an integer $s\in \mathbb {N}$, and data $x_s=\textbf{E}_{\textrm{I}}(s)$ obtained by conducting a primary experiment $\textbf{E}$ on *s*. The primary experiment yields the data from the domain $\mathcal {X}$ to be classified by the machine learning model $f:\mathcal {X}\rightarrow \mathcal {Y}$, which is trained to associate data from input domain $\mathcal {X}$ to labels $\mathcal {Y}$. The output of *f* is extended by an interpretable space $\mathcal {I}$. This interpretable space $\mathcal {I}$ can be either inferred using a post hoc interpretability method, or can be an inherent part of the model *f*. In this situation, given a data point $x_s$ with $f(x_s)=(h_s,y_s)\in \mathcal {I}\times \mathcal {Y}$, an explanation can be defined as a scientific hypothesis that connects the interpretable output $h_s$ with the sample *s* from which $x_s$ has been derived.

Now, the distinction between the real-world sample *s* and the data $x_s$ derived from it comes into play: For the hypothesis $\textbf{H}$ to be falsifiable, i.e., experimentally testable, the hypothesis needs to imply claims that predict the outcome of different *secondary experiments* $\textbf{E}_{\textrm{II}}(s,h_s),\textbf{E}_{\textrm{III}}(s,h_s),\dots$ to be conducted on *s*. Conducting further experiments on the identical sample *s* requires the sample to be re-identifiable through its unique integer identifier, which in medical applications will be a patient ID or a sample ID. As the notation indicates, the secondary experiments may be guided by the interpretable outcome $h_s$ obtained from *f*. Aligning the claims derived from $\textbf{H}$ with the observations from $\textbf{E}_{\textrm{II}}(s)$ corroborates or falsifies the hypothesis.

A specific example of the FXAI framework is shown in Fig. 3. Here, the neural network classifies tissue thin sections as *cancerous* vs. *cancer-free*, and along with the classification status of and input image, it provides a heatmap image that localizes those areas in the input image that are relevant for the specific output. The explanatory hypothesis in this example is relatively straightforward: We may claim that areas of high relevance, represented by high intensity in the heatmap image, localize tumor in the diseased samples. This is indeed a scientific hypothesis that can be tested in manifold ways by taking into account the vast knowledge about the hallmarks of cancer [19]. One could either stain commonly known oncogenes in the sample and investigate their co-localization with the activated regions in the heatmap image, or perform laser-capture microdissection and investigate the presence of oncogenic mutations in the regions. These are just two out of a potentially long list of experiments to test the *tumor-activation hypothesis*. The importance of this is that each of these experiments falsifies the hypothesis, and hence puts back into question the heatmap, along with the neural network from which the heatmap was generated. In other words, the explanatory hypothesis makes the neural network more vulnerable by adding additional points of potential experimental falsification.

The FXAI framework also provides an alternative to involve causality in explaining the output of AI models. As we have seen in the “Explainability approaches in artificial intelligence” section, counterfactual explanations can be scientific explanations in the sense that, at least in theory, they can be tested experimentally, although subject to the inherent difficulty to produce real-world objects that represent counterfactual data points. The FXAI framework allows to involve causal diagrams in a different manner, and one that avoids the problem of producing counterfactual objects. The idea is to simply identify the variables in the causal diagram with variables derived as an I-space. In a hypothetical example, this could look as follows: If we want to diagnose lung cancer based on tissue thin sections, a causal explanation could involve the causal diagram from Fig. 2. In order to involve the causal variable *tar deposits*, we can devise a neural network that produces *two* heatmaps for a given tissue sample, one that localizes tumor, and a second one that localizes tar deposits. If the tar-activation map displays tar deposits and the tumor-activation map displays tumor, one can hypothesize that the patient has developed cancer due to smoking (or other causes of tar deposits). This claim is also experimentally testable, as tar deposits have clear chemical characteristics. In other words, as long as causal diagrams involve experimentally measurable variables, they can potentially be used as experimentally testable causal explanations.

#### Explanation and causality

The two exemplars of vitamin C and lithium also demonstrate the relationship between causality and explanation in medicine. Their effectiveness is supported by evidence from RCTs as the widely accepted gold standard for validating therapeutic interventions [22], and randomized intervention in turn is commonly considered the gold standard of evidence for a cause-effect relationship between two variables [38]. RCTs can thus be identified with the second level on Pearl’s hierarchy of causation [38]. In other words, evidence for causality is a necessary criterion for any scientifically valid medical treatment. The case to be made from the example of scurvy is that unexplained causality lacks trustworthiness and is hence often not sufficient for sustainable translation into medical practice. In short, establishing causality from randomized intervention is necessary, but not sufficient for establishing trustworthiness, which in many cases requires explanation in addition.

#### Causality and explanation in biomedical AI

The role of explanation naturally translates from treatment measures to medical AI systems: if left unexplained, the adoption of AI systems in medical practice will foreseeably raise questions that put their legitimacy under debate. At first sight, treatment measures appear to differ substantially from diagnostic medical AI systems with respect to causality, since most diagnostic medical AI systems associate data with disease-related variables and thus lack intervention-based causality, locating them at the first level of Pearl’s hierarchy of causation. However, causality can be established by involving an AI system as part of the treatment decision in an RCT, which has, although rarely, been realized in few studies to date [27], moving such systems to the second level of the ladder of causation. Yet, despite standing on the firm grounds of causality, applying an unexplained AI system in medical routine may raise questions similar to the citrus remedy against scurvy.

## Scientific explanation, robustness, and trustworthiness

The integration of scientific explanation into machine learning calls for further discussion why scientific explanation fosters trustworthiness, which we discuss along the two aspects of robustness and the justification of inductive bias.

### The robustness of scientific explanations

As explained above, the introduction of a scientific explanation for an AI system is directly motivated by concerns of opacity, i.e., our limited understanding of how ML models succeed in prediction and what it is that they learn [3]. Scientific explanation improves upon current practice in XAI by subjecting the explanation of the ML model’s outputs to experimental testing. And as we have suggested above, this can lead to more trustworthy models. However, why believe explainability should be required for trustworthiness? Should reliability not be enough [13]?

We urge that considerations of trustworthiness and explainability are intimately intertwined in XAI, and that explanations on the ground of science have the potential to deliver actual trust*worthiness*, rather than unjustified trust. To see this, consider an important method for ensuring trust in one’s model or experiment, recognized under the name *robustness analysis* by various philosophers [28, 42]: In order to establish a scientific phenomenon, scientists vary experimental conditions, by using relevantly different materials, instruments, conditions, etc., to convince themselves that the phenomenon is not an artifact of particular conditions. For example, in the discovery of Brownian motion, Brown used several variations, including the use of living and dead organic materials, as well as inorganic materials, different containers, media, means of suspending the particles, and lighting, among others [42]. Similarly, for ensuring that one’s model in experimental analysis functions appropriately, it has to be checked whether the model’s predictions are robust, for instance, by varying its parameters, varying the model itself, varying the use of models in experimental analysis [4].

The concept of robustness analysis applies to any kind of scientific explanation that can be tested from different perspectives using different experiments. In particular, we can apply robustness analysis to scientific explanations of machine learning models, as realized in the FXAI framework. Here, the explicit distinction between the sample (or patient) *s* and the data $x_s$ derived from it allows us to use the same route to trust via robustness analysis within the FXAI framework: If a certain explanation $\textbf{H}$ emerges successfully after having tested it against implied claims $\textbf{C}_{\textrm{II}},\textbf{C}_{\textrm{III}},\dots$ tested through validation experiments, $\textbf{E}_{\textrm{II}}(s), \textbf{E}_{\textrm{III}}(s),\ldots$, then we have solid grounds for trusting both $\textbf{H}(s,h_s)$ and $f^+$. In contrast, if all we have is an empirical hypothesis instantiated by a machine learning model *f*, then we can typically not even *estimate* the relevant variations to be performed, in order to test whether the ML model has picked up on a real-world concept.

### Scientific explanations and machine learning theory

In order to facilitate machine learning models to pick up concepts from the real world, and for explanations to unveil these concepts, it is well understood that the underlying learning algorithm inevitably needs to involve suitable a priori assumptions in the form of its inductive bias. The inevitability of inductive bias naturally raises the *justification problem*: For a given learning problem, what is a legitimate choice of inductive bias? This problem is complicated due to the no-free-lunch (NFL) theorem [50], which states that when measuring classification performance in terms of off-training set (OTS) error, all learning algorithms, considered across the domain of all possible learning problems, exhibit equivalent performance. In particular, the NFL questions the use of cross-validation, and thus excludes cross-validation to formally justify the inductive bias of a specific learning algorithm.

The justification of inductive bias in the light of the NFL theorem has been addressed recently by Sterkenburg and Grünwald [46], arguing that that the injustifiability of inductive bias implied by the NFL results from a *data-only* view of machine learning, which is reflected by the common interpretation of the NFL that *all learning algorithms are equivalent*. Sterkenburg and Grünwald propose a different *model-dependant* interpretation of the NFL, by suggesting that *for any data-only learning algorithm, there exists a learning situation in which this algorithm does not perform well, while in this same situation, another data-only algorithm does perform well* [46].

This interpretation suggests that if algorithm choice is viewed as an integral part of machine learning, then mathematically provable learning-theoretic guarantees can justify the use of a specific algorithm. An example for such a learning theoretical guarantee provided in Sterkenburg and Grünwald [46] is that empirical risk minimization can be proven optimal under independent and identically distributed (i.i.d.) data. Yet, it is well known that i.i.d. is an unrealistic assumption that cannot be justified in many learning situations. Correspondingly, we here argue that provable learning-theoretic guarantees may in some situations be well-suited to characterize and hence justify inductive bias, but the problem of justification is merely shifted one step further: Now, the learning-theoretic guarantee needs justification, which, as illustrated for the case of i.i.d., may be problematic in practice.

To address the justification problem, we here suggest a *hypothesis-centric* view of machine learning by postulating that *the suitability of a learning algorithm for a specific learning situation can be assessed by experimentally testing an explanation of its output.* Here, an output may involve an $\mathcal {I}$-space as in the FXAI framework, and experimental testability refers to an *explanation* of the output rather than the output itself. Experimental testability of the explanation by definition identifies it as a hypothesis in the sense of the experimental sciences. It may rightly be argued that the justification of inductive bias is again shifted, namely to the identification of a testable explanation. If, however, for a given learning algorithm in a given learning setting, we identify an explanation, the implications of robustness analysis discussed in the “Scientific explanation, robustness, and trustworthiness” section apply.

The abstract postulate of hypothesis-centrism has immediate practical implications. In terms of the FXAI framework, it suggests to integrate the $\mathcal {I}$-space into the loss function. This step may not affect cross-validation performance, or even affect it negatively, but if the explanation is testable, one can assess potential improvement of explainability. As soon as the $\mathcal {I}$-space becomes part of the loss function in the sense of *interpretability-guided inductive bias* [33], optimizing towards interpretability becomes part of the training process, which means moving from post hoc to ante hoc interpretability.

## Case studies in current biomedical AI

With the widely recognized importance of explanation in medical AI, aspects of interpretation and explanation are an inherent part of numerous recent medical AI studies. While it is rarely discussed whether specific explanations are experimentally testable, this is sometimes implicitly the case. We here focus on studies in the field of computational pathology, where explanations in some cases relate to specific molecular alterations that can be tested experimentally within the tissue thin sections under consideration. Before reviewing the status of explainable AI systems in biomedicine, we provide a brief overview over the state-of-the-art of AI systems validated in RCTs.

### Assessment of AI decisions

With AI finding its way into medical practice, the first studies have been conducted recently that assess diagnostic AI systems as part of decision processes in RCTs. The state-of-the-art of evaluating AI in RCTs is provided in the scoping review by Han et al. in [18], which identifies 86 such studies conducted and published within the past 5 years. The authors of the survey find 81% of the studies reporting a positive outcome, while the average standard of the studies is rather moderate: They only involve a median of 359 patients and are largely not multi-centric, and only a few of the studies commit to reporting standards such as the CONSORT-AI statement. Yet, some recent studies point the way to high-quality outcomes. In Lång et al. [27], the authors conducted a population-based retrospective study involving more than 80,000 patients who underwent mammography screening in Sweden. The breast cancer incidence in the AI-diagnosed intervention group exhibited comparable breast cancer detection rates as the conventionally diagnosed control group, with a reported 44% workload reduction in the AI diagnosis. Another prominent RCT assessment of an AI system is reported in Lin et al. [29], where more than 15,000 patients were part of an AI-alert intervention in electrocardiogram (ECG) reading. The authors report that an AI-based ECG alert was associated with a significant reduction in all-cause mortality within 90 days compared to conventional ECG alert.

### Explainable tumor detection in computational pathology

We distinguish scientific explanations of two different types: First, several studies obtain explanations based on established post-hoc explanation approaches and connect the inferred interpretable outcome with explanations that meet the criteria of scientific hypotheses. A second approach, different from post hoc explanations, relies on inherently interpretable learning approaches. This stems from the popularity of so-called multiple instance learning [7, 32], which is one variant of weakly supervised machine learning. Multiple-instance learning addresses the problem of large image sizes in computational pathology: Tissue sections are commonly sized in the order of 1 cm$^2$, so that the microscopic image of an H& E stained tissue sample involves hundreds of millions or even billions of pixels. These whole-slide images are usually decomposed into smaller image patches of a fixed size of roughly 100 $\mu$m$^2$. Now, when trained on *cancer* vs. *cancer-free* samples, multiple instance learning utilizes annotations at the whole-slide level only, while classification at the patch level is left to an inference of the neural network. This inference is trained towards the assumption that a *cancer* slide contains at least one *tumor* patch, and *cancer-free* slides contain no *tumor* patch. The inferred patch labels provide an $\mathcal {I}$-space in the sense of the FXAI framework as follows: each patch is associated with one activation value, depending on how strongly it has been identified as a tumor patch. This activation map at the patch level can then be identified with the hypothesis that activation localizes tumor, which can be tested experimentally in various ways. Remarkably, for the examples identified here, the testability of the explanations is largely unexploited: The explanations are experimentally testable in theory, but the experimental testing has not been systematically conducted in practice. Furthermore, since multiple instance learning (MIL) infers these regions during training, they are subject to inductive bias, so that they can be regarded as explanation-guided.

A recent example where post hoc interpretability approaches, in this case the Grad-CAM approach [43], facilitate experimentally testable explanation has been provided by Försch et al. [14]. As the authors demonstrate, the inferred activation maps match established cellular patterns of anti-tumor immunity, which can in principle be tested using secondary experiments.

### Characterizing microsatellite status in colorectal cancer

We here use the recent work by Niehues et al. [36] on a computational pathology study for subtyping certain types of colon cancer to illustrate how scientific explanation can be accomplished in practice. The study employs weakly supervised learning to train two classifiers to subtyping colon carcinoma with clinical-grade performance. The first classifier differentiates microsatellite stable (MSS) from microsatellite instable (MSI) tumors, while the second determines the BRAF status as wildtype vs. mutated. For both classifiers, the authors use Grad-CAM for post-hoc interpretation to determine which regions are identified as classification-specific. We here focus on the model that performs MSS/MSI differentiation.

#### Deductive validation

Confirming earlier observations [16], the authors find that activation in MSS tissue is characterized by well-differentiated stroma-rich tissue areas, whereas activation in MSI tissue tends to indicate poorly differentiated tumor glands with immune-infiltrated stroma. These characterizations of MSS and MSI derived from the trained machine learning model in a post hoc manner can indeed each be regarded as falsifiable, i.e., experimentally testable, hypothesis: The cellular and molecular composition of stroma in the context of microsatellite instability has been investigated intensely [9], making it possible to experimentally test whether the Grad-CAM activated regions involve a certain amount of microsatellite-specific stroma tissue. Also, infiltrating immune cells can be identified with targeted staining techniques, or potentially also by laser-microdissection combined with transcriptome sequence or proteomics investigation. While the underlying hypotheses as well as the experimental assessment would need to be substantially sharpened, it is well conceivable to equip the machine learning model of the study by Niehaus et al. with deductive validation. In this direction, it is noteworthy that recent work [14] has introduced the systematic investigation of multistain approaches to localize different types of immune cells and their respective state, which may help to experimentally localize infiltrating immune cells.

In short, there is a plethora of experimental approaches to test the hypotheses derived from Grad-CAM activation. While these would at first be rather qualitative tests, it is generally conceivable, certainly with some substantial amount of scrutiny, to turn such experimental investigation into an objective, quantitative test.

#### From post hoc explanation to explanation-guided learning

Since the study by Niehues [36] confirms the previously established association of MSS/MSI status with stroma, immune infiltration, and tumor gland differentiation, these associations may be a reasonable basis for an explanation-guided machine learning model. Such an approach could start with identifying the tissue structures that the interpretation suggests are relevant: first, the localization of tumor [7, 32]; second, the localization of stroma [45]; third, the identification of infiltrating immune cells [44]; and fourth, the localization of tumor glands and an assessment of their degradation [6]. Although not all of the cited literature applies to colon tissue, these tasks appear solvable with state-of-the-art computational pathology approaches. Once the tissue structures relevant for the hypothesized explanation can be localized in a robust manner, one can train classifiers that utilize this presegmentation, which contains substantial amounts of domain knowledge that is required to test the explanation. Classifiers building on knowledge-based presegmentations will be inherently interpretable and testable, since the models for performing presegmentation can be tested using the experimental procedures sketched in the previous paragraphs.

The proposed procedure to move from post hoc explanation to explanation-guided learning is clearly speculative, but certainly conceivable and supported by state-of-the-art in computational pathology methodology. The question arises, what are the promised benefits to take the obviously steep technical, biological, and medical challenges towards explanation-guided learning? Clearly, the localized cellular and molecular mechanisms could be tested in various experimental ways, thus delivering the promise of robustness analysis and a gain trustworthiness. In fact, for each individual instance such a system is applied to, it can be experimentally tested whether the justifying explanation applies in this specific case. It must be stressed that there is no guarantee that a system whose training was guided by an explanation generalizes better to samples from cohorts unseen during training, or that it will achieve higher predictive accuracy. The compelling point towards trustworthiness is the testability of individual decisions, which can be conducted whenever doubt in a specific classification arises, for example, when an AI system is confronted with samples from a new cohort whose sample preparation was conducted under slightly varying laboratory conditions.

### Conclusions and discussion

We have made the case for scientific explanation in medical AI and demonstrated how it fosters trustworthiness, and how it relates to causality and the justification of inductive bias. While our focus has been on a conceptual perspective, the concepts are actionable in practice, as we exemplify in a concrete case in Supplement A. Some aspects deserve further conceptual investigation. In particular, we did not discuss the case of counterfactual causality, which constitutes the third level in the hierarchy of causation. Being based on the intervention-based *do*-calculus, counterfactual explanations are inherently experimentally testable, which deserves a dedicated separate discussion.

A natural limitation of scientific explanation is reached whenever the object under investigation changes its behavior when being aware of being predictable, which leads to a game-theoretic setting. This limits scientific explanation, as introduced here, to the experimental natural sciences, but excludes social sciences.

Another aspect not discussed here is the question of what makes a hypothesis a strong and useful explanation towards a specific medical application; also, some hypotheses may suggest more secondary experiments than others. We leave corresponding discussions of what constitutes “good” explanations for future work.

Finally, the gain in trustworthiness facilitated by scientific explanations has not yet been systematically exploited in practice. While some studies identify explanations that are explainable in principle, explanation-based deductive validation was not conducted in practice. Based on existing work in computational pathology, we have sketched how scientific explanation can be accomplished to distinguish microsatellite stable from microsatellite instable tumors in thin sections of colorectal tissue, and we elaborated on the costs and benefits of doing so. Based on our analogy to mechanisms of actions of drugs, we project that it will involve a case-by-case discussion of how much explanation a specific AI system needs. In some cases, AI-based decisions validated as part of RCTs may perform stable across many application settings over a long period of time. The need for explanation will become obvious in those cases where unexplained failures occur. As we argue, approaches to scientific explanation guide the way to trustworthy AI systems in such cases.

## Data Availability

No datasets were generated or analyzed during the current study.
